# Effect of a quality improvement program on compliance to the sepsis bundle in non-ICU patients: a multicenter prospective before and after cohort study

**DOI:** 10.3389/fmed.2023.1215341

**Published:** 2023-11-13

**Authors:** Gianpaola Monti, Emanuele Rezoagli, Angelo Calini, Alice Nova, Silvia Marchesi, Giovanni Nattino, Greta Carrara, Sergio Morra, Francesca Cortellaro, Monica Savioli, Federico Capra Marzani, Moreno Tresoldi, Paolo Villa, Stefano Greco, Paolo Bonfanti, Maria Grazia Spitoni, Sergio Vesconi, Pietro Caironi, Roberto Fumagalli, “Lotta alla Sepsi” Team Study Group, M. Aceti, M. Alborghetti, E. Beck, W. Bonalume, M. Bornaghi, S. Bosisio, E. Brusa, V. Canegrati, R. Capra, A. Cecchin, S. Ciccone, M. De Lucia, G. Erhan, C. Fabbri, L. Ferrante, N. Ferrari, S. Franchini, G. Gallotta, P. Galotti, P. Gavazzi, D. Grassi, M. Lattuada, C. Lorini, L. Manin, G. Marchesi, F. Margarito, F. Martini, M. Meo, G. Minoja, M. Ortolan, M. Padovani, M. Pecorino, A. Quaini, S. Quarteroni, D. Radrizzani, M. Ranzini, T. Santambrogio, V. Savojardo, A. Sciascera, G. Serra, M. Tajè, F. Tengattin, T. Tira, B. Tonolli, R. Traficante, M. Tresoldi, E. Varisco, M. Zarienato, C. Zeroli

**Affiliations:** ^1^Department of Anesthesia and Intensive Care, ASST GOM Niguarda Ca’ Granda, Milan, Italy; ^2^School of Medicine and Surgery, University of Milano-Bicocca, Monza, Italy; ^3^Department of Anesthesia and Intensive Care, Fondazione IRCCS San Gerardo dei Tintori, Monza, Italy; ^4^Intensiv och perioperativ vard, Skane Universitetssjukhus, Malmo, Sweden; ^5^Istituto di ricerche farmacologiche Mario Negri IRCCS, Ranica, Bergamo, Italy; ^6^Department of Anesthesia and Intensive Care, ASST Ovest Milano, Legnano, Italy; ^7^Azienda Regionale Emergenza Urgenza (AREU), Milan, Italy; ^8^Department of Anesthesia, Intensive Care and Emergency, Fondazione IRCCSC Ca' Granda-Ospedale Maggiore Policlinico, Milan, Italy; ^9^Department of Anesthesia and Intensive Care, Fondazione IRCCS Policlinico San Matteo, Pavia, Italy; ^10^Unit of General Medicine and Advanced Care, IRCCS San Raffaele Scientific Institute, Milan, Italy; ^11^Department of Emergency, ASST FBF - Sacco, Ospedale L. Sacco, Milan, Italy; ^12^Department of Anesthesia and Intensive Care, ASST Valle Olona, Ospedale Busto Arsitio, Busto Arsitio, Italy; ^13^Infectious Diseases, Fondazione IRCCS San Gerardo dei Tintori, Monza, Italy; ^14^ASST Bergamo Ovest, Ospedale di Treviglio e Caravaggio, Treviglio, Italy; ^15^Department of Anesthesia and Intensive Care, AOU S. Luigi Gonzaga, Università degli Studi di Torino, Orbassano, Italy

**Keywords:** quality improvement program, Sepsis, medical ward, emergency department, mortality

## Abstract

**Objective:**

Sepsis and septic shock are major challenges and economic burdens to healthcare, impacting millions of people globally and representing significant causes of mortality. Recently, a large number of quality improvement programs focused on sepsis resuscitation bundles have been instituted worldwide. These educational initiatives have been shown to be associated with improvements in clinical outcomes. We aimed to evaluate the impact of a multi-faceted quality implementing program (QIP) on the compliance of a “simplified 1-h bundle” (Sepsis 6) and hospital mortality of severe sepsis and septic shock patients out of the intensive care unit (ICU).

**Methods:**

Emergency departments (EDs) and medical wards (MWs) of 12 academic and non-academic hospitals in the Lombardy region (Northern Italy) were involved in a multi-faceted QIP, which included educational and organizational interventions. Patients with a clinical diagnosis of severe sepsis or septic shock according to the Sepsis-2 criteria were enrolled in two different periods: from May 2011 to November 2011 (before-QIP cohort) and from August 2012 to June 2013 (after-QIP cohort).

**Measurements and main results:**

The effect of QIP on bundle compliance and hospital mortality was evaluated in a before–after analysis. We enrolled 467 patients in the before-QIP group and 656 in the after-QIP group. At the time of enrollment, septic shock was diagnosed in 50% of patients, similarly between the two periods. In the after-QIP group, we observed increased compliance to the “simplified rapid (1 h) intervention bundle” (the Sepsis 6 bundle – S6) at three time-points evaluated (1 h, 13.7 to 18.7%, *p* = 0.018, 3 h, 37.1 to 48.0%, *p* = 0.013, overall study period, 46.2 to 57.9%, *p* < 0.001). We then analyzed compliance with S6 and hospital mortality in the before- and after-QIP periods, stratifying the two patients’ cohorts by admission characteristics. Adherence to the S6 bundle was increased in patients with severe sepsis in the absence of shock, in patients with serum lactate <4.0 mmol/L, and in patients with hypotension at the time of enrollment, regardless of the type of admission (from EDs or MWs). Subsequently, in an observational analysis, we also investigated the relation between bundle compliance and hospital mortality by logistic regression. In the after-QIP cohort, we observed a lower in-hospital mortality than that observed in the before-QIP cohort. This finding was reported in subgroups where a higher adherence to the S6 bundle in the after-QIP period was found. After adjustment for confounders, the QIP appeared to be independently associated with a significant improvement in hospital mortality. Among the single S6 procedures applied within the first hour of sepsis diagnosis, compliance with blood culture and antibiotic therapy appeared significantly associated with reduced in-hospital mortality.

**Conclusion:**

A multi-faceted QIP aimed at promoting an early simplified bundle of care for the management of septic patients out of the ICU was associated with improved compliance with sepsis bundles and lower in-hospital mortality.

## Introduction

Sepsis and septic shock are major challenges and economic burdens to healthcare globally, impacting millions of people around the world and representing significant causes of mortality. The estimated mortality rate ranges from 27% (for hospitalized patients) to 42% [for patients admitted to intensive care units (ICUs)] (1). As a time dependent condition, early identification and prompt appropriate care are essential to ensuring the best patient outcome.

Since its inception in 2002, the Surviving Sepsis Campaign (SSC) has aimed at increasing awareness of sepsis and improving the quality of care and the survival of patients with sepsis. The sepsis “bundles”—developed in parallel to the SSC guidelines—are useful tools to rapidly deliver diagnostic and therapeutic interventions in order to improve outcomes. The first sepsis bundle, published in 2004, included a “Sepsis Resuscitation Bundle” to be completed within the first 6 h of presentation and a “Sepsis Management Bundle” to be completed within the first 24 h (2). These initial bundles were changed in 2012 to a “3-h bundle” and “6-h bundle” (3). The most recent SSC bundle update was published in 2018. In this revision, the 3-h and 6-h bundles have been combined into a single “1-h bundle” (4). The five elements included in the “1-h bundle” were all confirmed in the most recent 2021 Surviving Sepsis Campaign (SSC) guidelines (5).

In patients with sepsis, adherence to resuscitation and treatment bundles has been demonstrated to be associated with improved outcomes (6, 7) and also with hospital cost savings (8). Similarly, in patients admitted to ICUs (6), the improvement in compliance with SSC bundles has been observed to be associated with a reduction in mortality.

A significant proportion of septic patients is diagnosed outside the ICU, either in the emergency department (ED) or in general wards (9–11). Indeed, the association between compliance with SSC bundles and improvement in clinical outcomes has not always been observed for septic patients treated out of the ICU (5), where resources are often limited and transferring evidence into clinical practice is not always simple.

Although a close correlation between guidelines implementation and survival has been confirmed (12), concerns have been raised about the effectiveness of single bundle elements, the correct timing of bundle application, and the superiority of protocolized care over an individualized treatment (13, 14).

In the last few years, quality improvement programs (QIPs) focused on sepsis resuscitation bundles have been instituted worldwide and are now recommended by 2021 SSC guidelines (5). A large number of qualitative improvement initiatives have been shown to be associated with improvements in mortality, reduction of length of stay, and saving in cost of care in the context of ICUs (15–18), as well as in medical wards (MWs) (19, 20). The Sepsis 6 bundle offers basic intervention for the treatment of sepsis within the first hour (21), and its application has been demonstrated to be independently associated with improved survival in a prospective observational study (22).

In 2011, a quality implementation program was introduced in Lombardy (Northern Italy) in order to improve the standard of care in sepsis diagnosis and treatment. The primary aim of this study was to examine the impact of a quality implementing program on the compliance of a “simplified rapid (1 h) intervention bundle” (the Sepsis 6 bundle – S6) applied in patients with severe sepsis or septic shock out of the ICU. The secondary aims of the study included the evaluation of S6 compliance at 3 h and during the entire study period; S6 compliance by baseline characteristics at the time of sepsis diagnosis; and the impact of single S6 procedures on in-hospital mortality. This study was part of the preliminary phase of the Lombardy region mandate for a sepsis care QIP, and it was employed as a test model before its widespread implementation throughout the Lombardy region.

## Methods

### Study design

This is a prospective multicenter before and after cohort study conducted as part of a QIP in the Lombardy region, Italy, including 12 EDs and 39 medical wards (MWs) of 12 academic and non-academic hospitals, accounting for 1,040 beds. ASST GOM Niguarda Ca′ Granda (Milan, Italy) is the coordinating center. The participating centers include ASST Ovest Milano Ospedale di Legnano (Legnano, Italy), Fondazione IRCCS Ca′ Granda Ospedale Maggiore Policlinico (Milan, Italy), Fondazione IRCCS Policlinico San Matteo (Pavia, Italy), IRCCS Ospedale San Raffaele (Milan, Italy), ASST Fatebenefratelli Ospedale L. Sacco (Milan, Italy), ASST Valle Olona Ospedale Busto Arsizio (Busto Arsizio, Italy), ASST Bergamo Ovest Ospedale Treviglio Caravaggio (Treviglio, Italy), ASST Papa Giovanni XXIII (Bergamo, Italy), ASST Sette Laghi Ospedale di Circolo Varese (Varese, Italy), ASST Brianza Ospedale di Desio (Desio, Italy), and ASST Lecco Presidio ospedaliero A. Manzoni (Lecco, Italy). Data on ED and MW, including the number of beds and volume of admission per year for each participating hospital, are presented in the Supplementary Materials.

Patients with a clinical diagnosis of severe sepsis or septic shock identified during the before and after study periods in the EDs or MWs were included. The attending physicians of each participating center screened patients admitted to the ED or the MW from May to November 2011 (before-QIP cohort) and from August 2012 to June 2013 (after-QIP cohort). The inclusion criteria were a confirmed or suspected infection and at least one laboratory or clinical sign of sepsis-induced organ dysfunction, including the following Sepsis-2 criteria (1): systolic blood pressure (SBP) ≤ 90 mmHg or mean arterial pressure (MAP) < 65 mmHg or drop >40 mmHg; serum lactate level ≥ 2 mmol/L or base deficit > −5 mEq/L; peripheral oxygen saturation (SpO_2_) < 90% or arterial partial pressure of oxygen to inspired oxygen fraction ratio (PaO_2_/FiO_2_) < 300; urine output (UO) < 0.5 mL/kg/h for more than 2 h or creatinine plasma concentration > 2 mg/dL or 1.5-folds the baseline value; bilirubin plasma concentration > 2 mg/dL or transaminase plasma level 2-folds the upper normal limit; prothrombin time (PT) international normalized ratio (INR) >1.5 or activated partial thromboplastin time (aPTT) > 60 s or platelet (PLT) counts <100 × 10^9^/L or < 0.5-fold the baseline value. The exclusion criteria were a surgical cause for hospital admission, an age lower than 18 years old, or pregnancy.

### Description of the QIP: educational and re-organizational intervention

The QIP started in the Lombardy region in 2011 with the following aims: (1) improving the quality of sepsis care; (2) implementing QI teams in the participating hospital; (3) measuring process and outcome markers; and (4) applying organizational interventions to facilitate the process of care (22).

The QIP included an educational and organizational intervention to implement resources available for the management of septic patients and to further overcome the barriers to its implementation. Both educational and re-organizational interventions were directed to EDs/MWs medical and nursing staff with the aim of increasing sepsis awareness and improving the standard of care for sepsis and septic shock. Our QIP aims at reaching compliance with S6 of ED/MW enrolled in the study equal to or greater than 80%. A local multidisciplinary QI team was involved in the educational and re-organizational interventions. Healthcare professionals (both physicians and nurses) from the participating centers attended a 5-h course that included frontal lectures as well as clinical case scenarios. The course was held by intensivists, microbiologists, infectious disease specialists, emergency physicians, and nurses according to their respective competences. The number of educational courses and the duration of the educational phase for each center were planned to include at least 80% of the staff.

We used a “waterfall model” of training to standardize the educational program among each participating center. The educational course was designed by the coordinating center (Niguarda Hospital, Milan, Italy), which was also responsible for the education of the local “QI trainers.” Local QI trainers were identified in each center and were involved in the educational process for their own hospital staff. Pocket cards and posters summarizing information on sepsis diagnosis and treatment were available in each hospital (Supplementary 1). For each participating center, local leaders (PI and area coordinator) were designed. The coordinating center organized meetings with local leaders before and after each study period. Before the QIP, hospital managers at each participating center were informed about the morbidity, mortality, and costs of sepsis, and the importance of the study was well described to ensure full institutional support. The PI created a local multidisciplinary team with representatives of all pertinent stakeholders (i.e., physicians and nurses from the ICU and infectious diseases, emergency, and internal medicine departments). Local leaders received monthly feedback about their center’s performance and distributed these results to all staff. The general coordinating center maintained continuous contact with the PI of each center through a mailing list. After the educational program, a survey was distributed to all PIs to monitor the quality of the local education program, verify the participation of all centers in the program, and know the PI’s evaluation of the main endpoints of the educational program: the presence of institutional support, the creation of a multidisciplinary team, and improvement in knowledge and hospital processes.

The diagnostic-treatment protocol focused on the Sepsis 6 bundle within 1 h after diagnosis and included the following items: (1) oxygen or ventilatory support, if needed; (2) blood cultures before the administration of antibiotics; (3) broad-spectrum appropriate anti-microbial therapy; (4) measurement of plasma lactate concentration; (5) administration of fluids in case of hypotension (i.e., mean arterial pressure ≤ 65 mmHg) or lactate concentration ≥ 4 mmol/L, as well as the administration of vasopressor agents (i.e., norepinephrine) in case of refractory hypotension; and (6) monitoring of diuresis (20).

In order to identify and face barriers to the implementation of a path toward the management of septic patients, an organizational logistic checklist (OLC) was provided to each participating center as a guide to identifying the resources/tools available locally for the management of septic patients (Supplementary Figure 1). OLC was completed by physicians of each participating center during both the before- and after-QIP periods.

Concrete goals and QI activities were discussed during the educational outreach sessions between QI teams and study coordinators. Regular meetings were organized to receive regular feedback from the participating centers and to keep the investigators up-to-date. A comprehensive list of meetings is provided in the Supplementary Methods.

A regional multidisciplinary advisory committee (MAC) was set up with the objective of defining an organizational model to implement the guidelines in clinical practice. This target was developed through the publication of regional decrees (n. 7,517, Atto identification.514, 5/08/2013) on the management of adult and maternal sepsis aimed at promoting an early simplified bundle for detection, risk stratification, and care in the management of septic patients out of the ICU.

### Study of intervention

Demographic and clinical characteristics at admission, timing of identification of sepsis (known or suspected), type of participating units (ED or MW), fulfillment of severe sepsis or septic shock criteria, infection site, compliance to S6 bundles, and other clinical interventions were recorded for the two cohorts of patients: the before-QIP cohort (enrolled from May to November 2011) and the after-QIP cohort (enrolled from August 2012 to June 2013). Data were recorded on a clinical checklist by physicians and nurses of each participating center (Supplementary Figure 2) and periodically entered into an electronic case report form (CRF) at the coordinating center. Data on the logistics of each center were registered at the beginning and at the end of the study. Data regarding hospital mortality were also recorded.

All the S6 bundle items, as well as other interventions, were assessed from the time when the patient was identified by the healthcare staff to fulfill Sepsis 2 criteria for severe sepsis or septic shock (time zero = T0) until intervention/s were performed. Compliance with S6 was then analyzed at 1 h, 3 h, and during the entire study period, lasting from the time of diagnosis and the implementation of the last procedure included in the S6 bundles. If any element was performed before T0, the patient was arbitrarily considered to have adhered to that element within the first hour.

### Definitions

According to the 2001 International Sepsis Definitions Conference (23) and the 2008 SSC (24), “severe sepsis” was defined as the presence of suspected infection and at least one sepsis-related organ dysfunction, whereas septic shock was defined as the presence of severe sepsis associated with the persistence of hypotension requiring vasopressor administration despite adequate fluid resuscitation or serum lactate ≥4 mmol/L.

### Statistical analysis

The distributions of categorical and binary variables are described using counts and proportions. Comparisons between groups are performed with Pearson’s chi-squared or Fisher’s exact test, as appropriate. The distributions of quantitative variables are described with the mean and standard deviation or median and interquartile range. Two-group comparisons are performed with the Wilcoxon rank-sum test. Unadjusted associations between educational programs and S6 compliance or hospital mortality are reported as relative risks. The association between adherence to every single intervention (applied either within the first hour or within the study period) and the reduction of the risk of death (hospital mortality) are reported as odds ratios.

In a subgroup analysis, we investigated the heterogeneity of the association between educational programs and (1) S6 compliance and (2) hospital mortality (reported as relative risks), across strata defined by: severity of the infection (severe sepsis/septic shock), ward of enrollment (ED/medical ward), hypotension (presence/absence), and serum lactate levels (≥ or < 4.0 mmol/L at enrollment). The heterogeneity of the associations was evaluated with the Breslow-Day test (23).

The chi-squared test was employed to evaluate differences between groups of patients managed after vs. before QIP. The analysis of differences in organizational characteristics of the participating centers before- and after-QIP was performed with McNemar’s test for paired data.

Finally, we estimated the association between educational programs and hospital mortality in multivariable analyses after adjusting for confounders. We used a logistic model with random intercepts corresponding to the enrolling center to account for the correlation among patients enrolled by the same center (24). The baseline characteristics included in the model were age, SOFA score, site of infection, and the presence of hypotension. An analogous model was used to estimate the adjusted association between compliance with the individual S6 procedures and hospital mortality.

No imputation of missing data was performed in the statistical analysis. Statistical analyses were carried out using R software.

### Ethical considerations

The study was supported by a Research Grant from Lombardy Region, Italy (Bando “Ricerca Innovativa,” 2011). Local ethics committee of each participating institution approved the study, after the approval of the Ethical Committee of the coordinating center (Delibera n. 1,137, 23 December 2010). Informed consent was waived, since the study was fully observational and no patient identifiers were collected. Patient information was reported anonymously, as sensitive information were expressed as an alphanumeric code.

The overall manuscript was structured according to the SQUIRE recommendations and checklist.^1^

## Results

### Baseline characteristics

A total of 467 patients were enrolled in the before-QIP period (from May 2011 to November 2011), whereas 656 patients were enrolled in the after-QIP period (from August 2012 to June 2013; Supplementary Figure 3). Approximately 39% of patients were enrolled in MWs, while 61% of patients were enrolled in EDs. No difference in demographic characteristics or site of infection between the two periods was observed (Table 1). Approximately 50% of patients had septic shock at the time of enrollment, similar between the two periods. In the before-QIP period, the prevalence of chronic renal failure (11.8% vs. 7.0%, *p* = 0.01) and baseline SOFA score [5 (3–7) vs. 5 (3–7); *p* = 0.02] were significantly higher than in the after-QIP period.

**Table 1:** Baseline characteristics of study population.

Characteristics	Before QIP	After QIP	Value of *p*
Patients, *n* (%)	467 (41.6)	656 (58.4)	**/**
Age – years, *n* = 1,088			
Mean (SD)	73.6 (13.9)	74.4 (13.7)	0.406
Median [Q1–Q3]	77 [66–84]	77 [67–84]	
Not available	17	18	
Female sex – N (%), *n* = 1,115	195/462 (42.2%)	279/653 (42.7%)	0.912
Not available	5	3	
Body weight – kg, *n* = 725			
Mean (SD) Median [Q1–Q3]	69 (14.7) 70 [60–78]	69.1 (15.3) 70 [60–80]	0.965
Female			
Mean (SD) Median [Q1–Q3]	63.6 (13.8) 60 [50.5–70]	63.1 (14.4) 60 [50–70]	0.718
Male			
Mean (SD) Median [Q1–Q3]	72.8 (14.1) 70 [65–80]	73.7 (14.5) 70 [63–80]	0.472
Not available	143	255	
Admission from – N (%), *n* = 1,123			
Emergency department	280/467 (60.0%)	403/656 (61.4%)	0.662
Medical ward	187/467 (40.0%)	253/656 (38.6%)	
Not available	0	0	
Surgery for source control – N (%), *n* = 1,105	52 (11.4%)	78 (12.0%)	0.863
Not available	13	5	
Primary site of infection – N (%), *n* = 1,106			0.115
Gastrointestinal	66 (14.4%)	62 (9.6%)	
Lung	163 (35.6%)	252 (38.9%)	
Central nervous system	7 (1.5%)	6 (0.9%)	
Soft tissues	28 (6.1%)	45 (6.9%)	
Urinary tract	115(25.1%)	186 (28.7%)	
Other site	16 (3.5%)	25 (3.9%)	
Unknown	63 (13.8%)	72 (11.1%)	
Not available	9	8	
Inclusion criteria			
Hypotension – N (%), *n* = 1,115	278 (60%)	378 (58.0%)	0.529
Not available	4	4	
Serum lactate ≥4 mmoL/L – N (%), *n* = 1,005	143 (38.3%)	215 (34.0%)	<0.001*
Not available	94	24	
Organ failures – N (%), *n* = 1,122	449 (96.1%)	635 (96.9%)	0.505
Not available	0	1	
Lactate – mmol/l, *n* = 757	5.6 (5.0)	5.1 (4.5)	0.127
Not available	196	170	
Diagnosis			
Severe sepsis, − N (%), *n* = 880	170/349 (48.7%)	273/531 (51.4%)	0.474
Septic shock – N (%), *n* = 880	179/349 (51.3%)	258/531 (48.6%)	
Not available	118	125	
Preexisting conditions – N (%)			
Liver disease, *n* = 1,009	42 (10.1%)	41 (6.9%)	0.083
Not available	53	61	
Respiratory disease, *n* = 1,010	74 (17.9%)	119 (20.0%)	0.453
Not available	53	60	
Chronic renal failure, *n* = 1,010	49 (11.8%)	42 (7.0%)	0.012*
Not available	53	60	
Immunodeficiency, *n* = 1,010	76 (18.4%)	105 (17.6%)	0.827
Not available	53	60	
Congestive or ischemic heart disease, *n* = 1,010	168 (40.6%)	207 (34.7%)	0.068
Not available	53	60	
SOFA score, *n* = 774	5.4 (2.9) 5 [3–7]	4.9 (2.7) 5 [3–7]	0.021*
Not available	145	204	

Liver disease was defined as histological diagnosis of cirrhosis with portal hypertension, previous gastrointestinal bleeding due to portal hypertension or previous episodes of hepatic insufficiency, encephalopathy or coma; respiratory disease was defined as a chronic restrictive, obstructive or vascular disease limiting normal activity and/or inducing hypoxia, hypercapnia, secondary polycythemia, pulmonary hypertension or mechanical ventilation dependency; chronic renal failure was defined as chronic dialysis; immunodeficiency was defined as an higher infection susceptibility due to medical therapy (steroids, chemotherapy, etc.) or diseases (leukemia, AIDS, etc.); congestive or ischemic heart disease was defined as a II-III-IV NYHA class or a ischemic cardiopathy. SOFA, Sequential Organ Failure Assessment. Sample size was reported for each described variable. *p < 0.05.

### Differences in S6 compliance between the before- and after-QIP period

**Figure 1:**
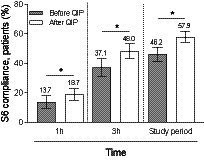
Patient compliance for the total S6 procedures within 1, 3 h and the total study period. Comparison between before and after QIP period. Histobars represent count (proportion) with 95% confidence interval. *p < 0.05 as reported in Tables 2A,B and Supplementary Table 2.

The number of patients compliant with total S6 procedures within 1 h significantly increased from 13.7% in the before-QIP period to 18.7% in the after-QIP period (*p* = 0.018; Figure 1; Table 2). Similar findings were observed when considering the compliance with S6 bundles within either 3 h (37.1% vs. 48.0%, respectively, *p* = 0.013; Figure 1; Supplementary Table 1) or the total study period (46.2% vs. 57.9%, *p* < 0.001; Figure 1; Table 2). When considering each single S6 procedure within 1 h, both patients receiving anti-microbial therapy and undergoing plasma lactate measurement significantly increased in the after-QIP period as compared to the before-QIP period (*p* = 0.026 and *p* < 0.001, respectively). Of note, among patients undergoing blood culture analysis, the median number of samples withdrawn increased from 2 [2–4] to 4 [2–4] (*p* < 0.001) after the educational period. Overall, the number of implemented S6 procedures increased after QIP (*p* = 0.018; Table 2A). Similar differences were observed between the before- and after-QIP cohorts during the entire study period (Table 2B).

**Table 2:** Primary outcome.

(A) Procedures applied within first hour	Before QIP	After QIP	Value of *p*
Evaluation of need for O_2_ /NIMV/MV – N (%), *n* = 861	348/393 (88.5%)	407/468 (870%)	0.548
Not available	74	188	
*time elapsed after enrollment – min, n = 755*	0 [0–0]	0 [0–10]	0.002*
Blood culture – N (%), *n* = 1,017	257/405 (63.5%)	415/612 (67.8%)	0.171
Not available	62	44	
*time elapsed after enrollment – min, n = 672*	3 [0–30]	7 [0–30]	0.210
*samples – N, n = 581*	2 [2–4]	4 [2–4]	<0.001*
Antibiotic therapy – N (%), *n* = 986	190/392 (48.5%)	332/594 (55.9%)	0.026*
Not available	75	62	
*time elapsed after enrollment – min, n = 522*	21.5 [0–43]	25.5 [0–45]	0.701
Lactate measurement – N (%), *n* = 1,054	225/433 (60.0%)	434/621 (69.9%)	<0.001*
Not available	34	35	
*time elapsed after enrollment – min, n = 659*	0[0–15]	0 [0–15]	0.458
Fluid administration – N (%), *n* = 1,047	194/436 (44.5%)	296/611 (48.4%)	0.230
Not available	31	45	
Urinary output measurement – N (%), *n* = 984	241(62.3%)	400 (67.0%)	0.147
Not available	80	59	
*time elapsed after enrollment – min, n = 641*	0 [0–20]	0 [0–20]	0.623
N of procedures adequately applied – N (%), *n* = 619			
0 procedure	6/256 (2.3%)	5/363 (1.3%)	0.018*
1 procedures	14/256 (5.5%)	16/363 (4.4%)	
2 procedures	32/256 (12.5%)	24/363 (6.6%)	
3 procedures	51/256 (20.0%)	50/363 (13.8%)	
4 procedures	58/256 (22.7%)	104/363 (28.7%)	
5 procedures	60/256 (23.4%)	96/363 (26.4%)	
All procedures (all 6) – N (%)	35/256 (13.7%)	68/363 (18.7%)	
Not available	211	293	
(B) Procedures applied within the entire study period^†^			
Evaluation of need for O_2_ /NIMV/MV – N (%), *n* = 1,113	445 (97.4%)	633 (96.5%)	0.514
Not available	10	0	
*time elapsed after enrollment – min, n = 826*	9.2 (93.4) 0 [0–5]	0.6 (268) 0 [0–15]	0.009*
Not available	64	188	
Blood culture – N (%), *n* = 1,117	389 (83.8%)	555 (85.0%)	0.658
*time elapsed after enrollment – min, n = 844*	15 [0–60]	15 [0–58.5]	0.800
Not available	3	3	
*samples – N, n = 768*	2 [2–4]	4 [2–4]	<0.001*
Not available	59	41	
Antibiotic therapy – N (%), *n* = 1,112	411 (89.5%)	614 (94.0%)	0.009*
Not available	8	3	
*time elapsed after enrollment – min, n = 899*	60 [20–120]	60 [15–120]	0.375
Not available	67	59	
Lactate measurement – N (%), *n* = 1,117	315 (67.9%)	554 (84.8%)	<0.001*
Not available	3	3	
*time elapsed after enrollment – min, n = 806*	0 [0–30]	0 [0–20]	0.791
Not available	31	32	
Fluid administration – N (%), *n* = 1,047	194 (44.5%)	296 (48.4%)	0.230
Not available	31	45	
Urinary output measurement – N (%), *n* = 1,109	372 (80.7%)	554 (85.5%)	0.041*
Not available	6	8	
*time elapsed after enrollment – min, n = 801*	10 [0–45]	10 [0–45]	0.972
Not available	74	58	
N of procedures adequately applied – N (%), *n* = 1,066			
0 procedures	0/429 (0.0%)	0/637 (0.0%)	<0.001*
1 procedure	2/429 (0.5%)	0/637 (0.0%)	
2 procedures	4/429 (0.9%)	6/637 (0.1%)	
3 procedures	39/429 (9.1%)	22/637 (3.5%)	
4 procedures	50/429 (11.7%)	63/637 (9.9%)	
5 procedures	136/429 (31.7%)	177/637 (27.8%)	
All procedures (all 6) – N (%)	198/429 (46.2%)	369/637 (57.9%)	
Not available	38	19	

Analysis of adherence to the sepsis’ six procedures (applied either within the first hour A) or within the study period B) between the two study groups (control period vs. intervention period). ^†^included patients in which procedures have been applied within the 1st h, and included in the table above. *p < 0.05.

### S6 Compliance stratified by characteristics at admission

**Figure 2:**
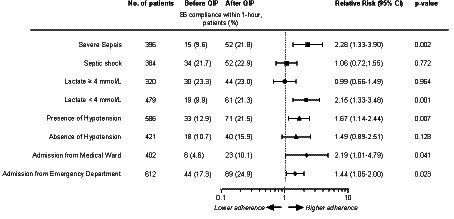
Forrest Plot of adherence to sepsis six bundle within 1-h stratified by baseline characteristics in the after vs. before QIP period. Dashed line describes the RR = 1. Missing data are as follows: 1. Severe sepsis and septic shock: Before QIP, *n* = 153; After QIP, *n* = 190; 2. Lactate levels: Before QIP, *n* = 146; After QIP, *n* = 178; Presence or absence of hypotension: Before QIP, *n* = 42; After QIP, *n* = 74; Location of admission: Before QIP, *n* = 38; After QIP, *n* = 71.

We then analyzed the primary outcome by study groups, including either the presence of severe sepsis or septic shock, the site of enrollment (either in ED or MW), the presence or absence of hypotension, and the presence or absence of serum lactate ≥4.0 mmol/L, both at the time of enrollment (Supplementary Tables 2, 3; Figure 2). The adherence to the application of the S6 bundle increased in patients with severe sepsis without shock (*p* = 0.002), in patients with serum lactate <4.0 mmol/L at the time of enrollment (*p* = 0.007), and in patients with hypotension at the time of study enrollment (*p* = 0.007; Supplementary Tables 2, 3). Adherence to S6 increased in both patient groups admitted to the ED (0.023) and MW (0.041; Supplementary Table 2). The effectiveness of the educational intervention was observed between the two periods in relation to the severity of organ failures, as denoted by the SOFA score (Supplementary Table 2). Similar findings were observed when differences in S6 compliance were investigated within the entire study period, with the exception of the SOFA score, as S6 adherence increased significantly across the three increasing groups of SOFA score severity (Supplementary Table 3).

### Hospital mortality

Hospital mortality in the entire study population showed a trend toward a lower rate after QIP as compared to before QIP (32.4% vs. 37.9%, respectively, *p* = 0.062; Supplementary Table 4). Indeed, after QIP, hospital mortality significantly decreased in patients with severe sepsis without shock (38.2% vs. 25.4%, *p* = 0.005, relative risk 0.66, 95% CI 0.50–0.88), serum lactate <4.0 mmol/L (36.4% vs. 24.5%, *p* = 0.003, relative risk 0.67, 95% CI 0.52–0.87), hypotension (47.5% vs. 36.1%, *p* = 0.004, relative risk 0.76, 95% CI 0.63–0.91), and higher levels of SOFA score (55.0% vs. 38.3%, *p* = 0.014, relative risk 0.70, 95% CI 0.52–0.93). No difference in hospital mortality was observed in patients with septic shock, serum lactate ≥4.0 mmol/L, no presence of hypotension, and a lower level of SOFA (Supplementary Table 5).

After adjustment for confounders, including age, SOFA score, site of infection, and the presence of hypotension, the QIP appeared to be independently associated with a significant improvement in hospital mortality (Table 3). Among the single S6 procedures applied within the first hour of sepsis diagnosis, compliance for blood culture (*p* < 0.001), antibiotic therapy (*p* = 0.046), and assessment of urinary output (*p* = 0.034) were significantly associated with a reduction in in-hospital mortality. Furthermore, the increasing number of S6 procedures was significantly associated with reduced in-hospital mortality (*p* for trend = 0.031; Supplementary Table 6A). The association between bundle compliance with the different S6 procedures and in-hospital mortality is shown in Supplementary Table 6B. In an adjusted multivariable model, an increasing number of S6 procedures trended toward a lower in-hospital mortality rate (*p* = 0.072; Table 4). Blood culture and lactate measurement were negatively associated, whereas the measurement of urinary output was positively associated, with in-hospital mortality (Table 5).

**Table 3:** Adjusted analyses.

Variable	OR (95% CI)	Value of *p* (Wald test)
Age (continuous, in decades)	1.43 (1.23–1.66)	<0.001*
Sex (M vs. F)	1.11 (0.78–1.57)	0.560
Site of the infection (abdomen vs. UTI)	0.97 (0.53–1.80)	0.124
Site of the infection (other vs. UTI)	0.63 (0.22–1.81)	
Site of the infection (lung vs. UTI)	1.99 (1.30–3.03)	
Site of the infection (soft tissue vs. UTI)	3.58 (1.67–7.66)	
Site of the infection (unknown vs. UTI)	1.31 (0.73–2.35)	
Hypotension (Yes vs. No)	1.49 (1.02–2.18)	0.365
SOFA (continuous)	1.22 (1.13–1.30)	<0.001*
After vs. Before QIP	0.64 (0.46–0.91)	0.012*

Analysis of association of After vs. Before QIP on hospital mortality after adjustments for clinically relevant variables, *n* = 737. *p < 0.05.

**Table 4:** Adjusted analyses.

Variable	OR (95% CI)	Value of *p* (Wald test)
Age (continuous, in decades)	1.40 (1.20–1.64)	<0.001*
Sex (M vs. F)	1.06 (0.73–1.54)	0.759
Site of the infection (abdomen vs. UTI)	0.93 (0.48–1.79)	0.280
Site of the infection (other vs. UTI)	0.53 (0.17–1.72)	
Site of the infection (lung vs. UTI)	1.94 (1.23–3.07)	
Site of the infection (soft tissue vs. UTI)	3.66 (1.66–8.07)	
Site of the infection (unknown vs. UTI)	1.07 (0.56–2.04)	
Hypothension (Yes vs. No)	1.30 (0.86–1.96)	0.211
Serum lactate ≥4 mmoL/L (Yes vs. No)	1.51 (1.01–2.26)	<0.061
Serum lactate ≥4 mmoL/L (Unknown vs. No)	1.51 (0.77–2.98)	
SOFA (continuous)	1.22 (1.13–1.32)	<0.043*
Number of completed Sepsis Six (continuous)	0.83 (0.67–1.02)	0.231

Analysis of association of number of applied S6 on hospital mortality after adjustments for clinically relevant variables, *n* = 637. *p < 0.05.

**Table 5:** Adjusted analyses.

Variable	OR (95% CI)	Value of *p* (Wald test)
Age (continuous, in decades)	1.35 (1.16–1.58)	<0.001*
Sex (M vs. F)		0.601
Site of the infection (abdomen vs. UTI)	0.97 (0.50–1.87)	0.212
Site of the infection (other vs. UTI)	0.58 (0.18–1.86)	
Site of the infection (lung vs. UTI)	1.95 (1.23–3.08)	
Site of the infection (soft tissue vs. UTI)	3.86 (1.75–8.51)	
Site of the infection (unknown vs. UTI)	1.10 (0.57–2.10)	
Hypothension (Yes vs. No)	1.20 (0.79–1.82)	0.374
Serum lactate ≥4 mmoL/L (Yes vs. No)	1.60 (1.08–2.39)	0.107
Serum lactate ≥4 mmoL/L (Unknown vs. No)	1.27 (0.63–2.57)	
SOFA (continuous)	1.22 (1.13–1.31)	<0.001*
Ventilation – SS1 (Yes vs. No)	1.05 (0.34–3.26)	0.908
Blood culture – SS2 (Yes vs. No)	0.56 (0.34–0.91)	0.020*
Antibiotic therapy – SS3 (Yes vs. No)	0.75 (0.36–1.55)	0.469
Lactate measurement – SS4 (Yes vs. No)	0.50 (0.28–0.91)	0.025*
Fluid administration – SS5 (Yes vs. No)	0.88 (0.38–2.04)	0.777
Urinary output measurement – SS6 (Yes vs. No)	1.89 (1.02–3.51)	0.040*

Analysis of association of individual S6 on hospital mortality after adjustments for clinically relevant variables, *n* = 637. *p < 0.05.

Finally, no differences in the management of patients between the before-QIP and the after-QIP periods were observed, with the exception of central venous catheter placement. Indeed, we observed a higher availability of sepsis teams or rapid response system teams after the QIP.

Differences regarding the optimization of organizational and logistics resources during the two periods are reported in Supplementary Tables 7, 8.

## Discussion

The main findings of this multicenter prospective before and after cohort study, conducted out of ICUs in 12 academic and non-academic hospitals in the Lombardy region, Italy, are as follows:

- The overall compliance with the S6 bundles increased after QIP within 1 h, within 3 h, and during the entire study period. When considering each single S6 procedure, the proportion of patients receiving anti-microbial therapy, undergoing blood culture analysis, and plasma lactate measurement significantly increased after QIP.
- After the stratification of patients by specific subgroups, we observed a higher adherence to the application of the S6 bundle after QIP in patients with severe sepsis without shock, in patients with serum lactate <4.0 mmol/L, in patients with hypotension at the time of enrollment, and in patients with a higher SOFA score. Adherence to S6 increased after QIP in both patient groups admitted to the ED and MW.
- After adjustments for clinically relevant variables, we observed a significant association between QIP and hospital mortality. When considering each S6 procedure applied, blood culture analysis and plasma lactate measurement were observed to be associated with decreased in-hospital mortality, whereas urinary output measurement was associated with increased in-hospital mortality.

Our results show that the implementation of educational and organizational interventions was associated with a significant improvement in compliance with guidelines for the management of patients with severe sepsis or septic shock outside ICUs, according to previous literature (18–20, 25, 26). Of note, although this was not our primary outcome, hospital mortality in the entire study population after QIP was lower than before QIP and reached a trend toward statistical significance. A relative risk reduction for hospital mortality was observed in patients with severe sepsis without shock, serum lactate <4.0 mmol/L, hypotension, and a higher SOFA score after the QIP. In an adjusted multivariable model, the implementation of QIP appeared to be independently associated with a significant reduction of in-hospital mortality, whereas no association was observed with an increasing number of S6 procedures after adjustments for clinically relevant variables. Among each single S6 procedure, compliance with blood culture and antibiotic therapy was significantly associated with reduced in-hospital mortality.

Although it is difficult to demonstrate a causal relationship between educational intervention and reduced mortality, one could argue that this might be the case based on several considerations. First, a reduction in in-hospital mortality after QIP was found for the same subgroups of patients (severe sepsis without shock, lactate <4.0 mmol/L, hypotension, and higher SOFA), in which a significant increase in S6 bundle compliance was observed. In the septic shock population, we did not observe either an increase in compliance or a significant reduction in in-hospital mortality. In our study, the educational program was directed to the “front line actor” in suspecting and managing the early phase of sepsis (i.e., ED/MW clinicians and nurses) with the aim of increasing sepsis awareness and guaranteeing prompt diagnosis and treatment as the rapid response team (RRT) may be alerted late and a sepsis team is not always present in the hospital. Otherwise, the management of the septic shock is the task of the intensivist. In this condition, once cardiovascular failure and hypoperfusion have developed, the outcome is probably less dependent on the delivery of the resuscitation bundle. This could be the reason why, in this context, compliance with the sepsis bundle did not show an increase after the QIP. Then, the significant increase in compliance of some individual bundle elements may directly impact the outcome. The prescription of broad-spectrum antibiotics and the measurement of plasma lactate as important signs of potential cardiovascular failure may contribute, respectively, to appropriate source control of the infection and to the early detection (and sequential treatment) of hypoperfusion. Plasma lactate measurement and blood culture analysis are associated with decreased in-hospital mortality after adjustments for clinically relevant variables. This finding underlines the importance of the early diagnosis and treatment of both septic sources and hemodynamic impairment. In adjusted analysis, we observed an association between urinary output measurement and increased mortality. This result, though unexpected, may suggest that urinary output was likely early monitored in more severe patients or in patients affected by comorbidities (i.e., chronic cardiac failure and chronic renal failure).

As compared with previous literature, our research presents some novelties.

Several studies have addressed how QIP based on sepsis bundles affects patient outcomes both in the ICU (16, 17) and in the MW (19, 20) through retrospective analyses. In this case, sepsis may be underdiagnosed or over diagnosed because patients were selected through hospital chart review, thereby affecting the final results. To the best of our knowledge, this is the first multicenter prospective study that evaluates the effect of a sepsis care implementation program using a “1-h simplified bundle” (Sepsis 6) outside ICUs.

It is also important to note that data on the mortality of sepsis are available in the clinical settings of the ED or ICU (6, 27, 28), whereas inadequate data are available for general medical wards, where, to date, no study has investigated this issue in Italy. Our study was performed in a group of hospitals voluntarily engaged in the Q1P program, but we can suppose that our results may be generalized to a huge part of the North Italian healthcare system considering the different affiliations (academic vs. non-academic hospitals) and the wide case volume of the participating hospitals.

Our study was conducted in a fully industrialized region with a high demand for hospital assistance. In this context, the early recognition and appropriate treatment of sepsis, as shown by our study results, would potentially lead to improved hospital length of stay, improved outcomes, and reduced public expenses. Our quality improvement program included both educational and organizational interventions, and it was aimed at facilitating the delivery of the evidence-based resuscitation procedures proposed by the SSC guidelines during the study period (29) and summarized in the S6 bundle (21). The educational intervention of our QIP embodies most of the strengths of this study. Our QIP was directed to healthcare professionals in the ED and MW, where the majority of septic patients are treated (9–11). Patients admitted to MWs are usually elderly and affected by chronic comorbidities, thereby being at high risk of developing infections. In this category of patients, classic signs and symptoms of sepsis could be blunted due to the high rate of multi-morbidity and poly-pharmacotherapy (30). Such clinical contexts, along with the lack of invasive monitoring and an “intensive care environment,” can delay the identification of patients at risk. Of note, the simplified 1-h bundle tool very likely appeared sufficiently handy and simple to be managed out of the ICU by non-critical care physicians as well as nurses. Furthermore, in the participating centers, the availability of an early warning score system was implemented after QIP, further supporting the importance of early warning score systems (i.e., q-SOFA) to alert for critical conditions (5). For these reasons, patients treated out of the ICU represent a proportionally more vulnerable population, which may benefit from an augmented sepsis early warning system. The healthcare professionals’ training based on the “waterfall method” and the standardized educational program proposed in our study could facilitate the rapid identification and intervention of sepsis, avoiding the evolution of septic shock and multiorgan failure.

The educational initiative proposed in our study focused on early and adequate adherence to the resuscitation bundle, which has resulted in a more pronounced mortality reduction than compliance with the organizational bundle (31). The attention to the 1-h Sepsis 6 bundle of our educational program makes it up-to-date and in line with current guidelines. A more recent trial has demonstrated that delays in the delivery of resuscitation bundles, even when they did not exceed 3 h, were associated with a significant increase in hospital mortality (32), supporting the timeframe of 1 h, proposed in the S6 bundles (21). Our study supports the validity of the 1-h S6 bundle as an efficient tool to implement compliance with the new resuscitation bundle of SSC guidelines, as the S6 bundle has anticipated some of the bundles of the 2018 SSC guidelines (4). Of note, our investigation was focused not only on the impact of the overall S6 bundle but also on the impact of compliance with each single S6 item on outcomes. Moreover, we also evaluated how each single item was administered to the patients (i.e., the number of sets of blood cultures obtained and the dose of fluids). This highlights how each element should be emphasized during the bundle application. It is likely that this approach may have a positive impact on personnel education and, consequently, on compliance (primary aim) and mortality (secondary aim). As an example, we observed a significant association between adherence to a single intervention (culture execution and administration of antibiotic therapy) in the first hour and the reduction of the risk of death, as previously reported (33). This finding emphasizes once again that the critical importance of delivering broad-spectrum antimicrobials to patients with sepsis or septic shock should be considered as an emergency action, as the early administration of appropriate antimicrobials is one of the most effective interventions to reduce mortality in patients with sepsis (12, 34, 35).

The comparison of our results with other studies is limited by the poor literature focusing on the 1-h S6 bundle. Most of these studies explored the 3-h bundle of the SSC and did not evaluate the impact of it at 1 h. Some research studies evaluated the early management bundle in patients with sepsis or septic shock: antibiotics and blood culture, fluids within 3 h, and re-evaluation of lactate levels within 6 h in US hospitals (36, 37).

Baseline S6 compliance at 1 and 3 h was relatively low: 13.7 and 37.1%. This may be due to the limited compliance with S6 in MWs, where resources are limited and awareness of time dependency is still poor. Similar results have been reported in recent large studies (8, 38–40). After the QI program, we observed a significant increase in S6 compliance both in MWs and EDs, although we did not reach the targeted compliance (≥ 80%).

Some considerations should be kept in mind. First, the compliance reported in our study was facilitated by the concomitant publication of the regional recommendation on sepsis within the regional decree (n. 7,517, Atto identification 514, 5/08/2013). The Lombardy region program further promoted and supported the QI program by funding the current study. Second, compliance with the 3- and 6-h bundle was required and consequently evaluated only for patients in whom a protocol was initiated, thereby likely overestimating the effective adherence to the QIP. On the other side, we did not include patients with sepsis or septic shock in critical care settings, which are characterized by better compliance as compared to the ED and general wards (41). A great portion of our patients were treated in MWs, which had an impact on the overall compliance and mortality rate as reported in the literature (36). It is also important to note that the adherence to S6 bundles in patients admitted to MWs almost doubled from the before- to the after-QIP period (from 26.6 to 48.2%, *p* < 0.001) when considering the entire study period. This may suggest a difficulty in the rapidity of bundle applications in the general ward setting rather than in their application *per se*.

The organizational intervention of our QIP represents the second peculiar aspect of our study. The QIP was sustained by a hospital multidisciplinary team responsible for communication, education, and data collection. We proposed a well-planned strategy based on an organizational logistic checklist (OLC) to be used as a guide to face the main barriers to septic patient management in each hospital. The improvement in the process of care may be associated with the resolution of many logistic barriers, suggesting a possible key role of “re-organization” in quality improvement programs.

Our study has several limitations. First, our data date back to 10 years. This delay in data communication has two main implications. Our study focused on the identification and treatment of patients with severe sepsis and septic shock during the time of the Sepsis-2 consensus definitions (29) before the publication of the Sepsis-3 definitions (42). The new definition of sepsis might have increased the specificity of our cohort for mortality, as a higher SOFA score is associated with an increased mortality risk (38). Then, the emerging clinical challenges, as well as the evolution of organizational models of both EDs and MWs, especially after the COVID-19 pandemic, might modify the impact of a QIP in these settings. Second, we were not able to evaluate a possible association between bundle compliance and case volume or organizational resources. Third, the time of enrollment was arbitrarily considered as the moment when the clinical team suspected sepsis, taking into account that the appropriate detection of signs and symptoms of sepsis onset may be difficult, especially in MW patients. Furthermore, data were recorded by attending physicians on duty during daily clinical practice. In some situations (i.e., high load of work and emergency conditions), patient care takes priority over detailed data recording, leading to missing data. No imputation of missing values was performed in the statistical analysis. Fourth, patients with septic shock were identified retrospectively based on the data collected in the CRF. Due to a relatively high percentage of patients showing missing values for at least one of the variables used for the categorization and in the absence of any missing imputation protocol applied, some patients with septic shock could be included only as patients with sepsis, and some of the patients included in the study could not have been included in the subgroup analysis on potential differences between patients with sepsis without shock and those with shock. Fifth, the before and after design is an inherent limitation of the study since it prevents causal interpretation. Finally, even if our study, conducted in the Lombardy region (Northern Italy), can be generalized to the Lombardia healthcare system within the Italian country, differences may be reported in comparison with different European and extra-European healthcare systems.

## Conclusion

In conclusion, the findings of this study demonstrate that a multi-faceted quality improvement program was successful in improving sepsis diagnosis and treatment in EDs and MWs, as evidenced by a significant increase in compliance with the 1-h S6 bundle. The performance of a QIP appeared to be independently associated with a reduction in hospital mortality in multivariate analysis, especially in specific subgroups of patients. Taken together, these results suggest that education interventions offer the best available tool for implementing guidelines adherence and improving outcomes even outside of ICUs. For these reasons, similar efforts should be encouraged to increase sepsis warnings, improve the quality of care, and target the best patient outcomes.

## Data availability statement

The datasets presented in this article are not readily available because these data are under copyright of Regione Lombardia – so data might be available upon reasonable request to Regione Lombardia. Requests to access the datasets should be directed to Regione Lombardia – roberto.fumagalli@unimib.it.

## Ethics statement

The studies involving human participants were reviewed and approved by the Scientific Ethics Committee of the coordinating center (President Bruno Mario Cesana) Azienda Ospedaliera Niguarda Ca’ Granda on March 21, 2011 within the Project “Ricerca innovativa – Lotta alla sepsi" included in “Delibera n. 1137 del 23 Dicembre 2010” titled “Determinazione in ordine a progetti afferenti all’ambito della ricerca innovativa” of Regione Lombardia. Local ethics committee of each participating institution approved the study, after the approval of the Scientific Ethics Committee of the coordinating center Azienda Ospedaliera Niguarda Ca’ Granda. Written informed consent to participate in this study was waived in accordance to the Scientific Ethics Committee of the coordinating center, since the study was fully observational and no patient identifiers were collected. Patient information was reported anonymously, as sensitive information was expressed as an alphanumeric code.

## Author contributions

GM, ER, and RF: conceptualization, methodology, project administration, and supervision. ER: software, data curation, and visualization. GM, ER, and PC: validation. ER, SM, GN, and GC: formal analysis. RF: funding acquisition and resources. GM, CP, AN, and ER: writing—original draft preparation. GM, ER, AC, AN, SM, GN, GC, SM, FC, MS, FCM, MT, PV, SG, PB, MGS, SV, PC, and RF: writing—review and editing. All authors contributed to the article and approved the submitted version.

## “Lotta alla Sepsi” Team Study Group investigators

Aceti M., Alborghetti M., Beck E., Bonalume W., Bornaghi M., Bosisio S., Brusa E., Canegrati V., Capra R., Cecchin A., Ciccone S., De Lucia M., Erhan G., Fabbri C., Ferrante L., Ferrari N., Franchini S., Gallotta G., Galotti P., Gavazzi P., Grassi D., Lattuada M., Lorini C., Manin L., Marchesi G., Margarito F., Martini F., Meo M., Minoja G., Ortolan M., Padovani M., Pecorino M., Quaini A., Quarteroni S., Radrizzani D., Ranzini M., Santambrogio T., Savojardo V., Sciascera A., Serra G., Tajè M., Tengattin F., Tira T., Tonolli B., Traficante R., Tresoldi M., Varisco E., Zarienato M., Zeroli C.

## Footnotes

^1^ https://www.equator-network.org/reporting-guidelines/squire/
