# Supplementary material for: Effect of a quality improvement program on compliance to the sepsis bundle in non-ICU patients: a multicenter prospective before and after cohort study
Source: Front Med (Lausanne). 2023 Nov 13;10:1215341. doi: 10.3389/fmed.2023.1215341 (PMC10680451; doi:10.3389/fmed.2023.1215341)
Supplement: Supplementary file 1 [file Data_Sheet_1.docx]

**Supplemental Material**

**A quality improvement program to improve sepsis bundle compliance in non-ICU patients: a multicenter prospective before and after cohort study.**

Monti Gianpaola, MD* Rezoagli Emanuele, MD PhD* Calini Angelo, MD Nova Alice, MD Marchesi Silvia, PhD Nattino Giovanni, PhD Carrara Greta, PhD Morra Sergio, MD Cortellaro Francesca, MD Savioli Monica, MD Capra Marzani Federico, MD Tresoldi Moreno, MD Villa Paolo, MD Greco Stefano, MD Bonfanti Paolo, MD Spitoni Maria Grazia, MD Vesconi Sergio, MD Caironi Pietro, MD Fumagalli Roberto, MD on behalf of “Lotta alla Sepsi” Team Study Group.

*Joint co-first authorship

Monti Gianpaola

Department of Anesthesia and Intensive Care, ASST GOM Niguarda Ca’ Granda, Milan Italy

Email: [gianpaola.monti@ospedaleniguarda.it](mailto:gianpaola.monti@ospedaleniguarda.it)

Rezoagli Emanuele

School of Medicine and Surgery, University of Milano-Bicocca, Monza, Italy

Department of Anesthesia and Intensive Care, Fondazione IRCCS San Gerardo dei Tintori, Monza, Italy

Email: [emanuele.rezoagli@unimib.it](mailto:emanuele.rezoagli@unimib.it)

Calini Angelo

Department of Anesthesia and Intensive Care, ASST GOM Niguarda Ca’ Granda, Milan Italy

Email: [angeloraffaele.calini@ospedaleniguarda.it](mailto:angeloraffaele.calini@ospedaleniguarda.it)

Nova Alice

School of Medicine and Surgery, University of Milano-Bicocca, Monza, Italy

Email: [a.nova6@campus.unimib.it](mailto:a.nova6@campus.unimib.it)

Marchesi Silvia

Intensiv och perioperativ vard, Skane Universitetssjukhus, Malmo, Sweden

Email: [silvia.marchesi@skane.se](mailto:silvia.marchesi@skane.se)

Nattino Giovanni

Istituto di ricerche farmacologiche Mario Negri IRCCS, Ranica (BG), Italy

### Email: [giovanni.nattino@marionegri.it](mailto:giovanni.nattino@marionegri.it)

Carrara Greta

Istituto di ricerche farmacologiche Mario Negri IRCCS, Ranica (BG), Italy

Email: [greta.carrara@marionegri.it](mailto:greta.carrara@marionegri.it)

Morra Sergio

Department of Anesthesia and Intensive Care, ASST Ovest Milano, Legnano, Italy

Email: sergio.morra@ao-legnano.it

Cortellaro Francesca

Azienda Regionale Emergenza Urgenza (AREU), Milan, Italy

Email: [f.cortellaro@areu.Lombardia.it](mailto:f.cortellaro@areu.Lombardia.it)

Savioli Monica

Department of Anesthesia, Intensive Care and Emergency, Fondazione IRCCSC Ca' Granda-Ospedale Maggiore Policlinico, Milan, Italy

Email: monica.savioli@policlinico.mi.it

Capra Marzani Federico

Department of Anesthesia and Intensive Care, Fondazione IRCCS Policlinico San Matteo, Pavia, Italy

Email: f.capramarzani@smatteo.pv.it

Tresoldi Moreno

Unit of General Medicine and Advanced Care, IRCCS San Raffaele Scientific Institute, Milan, Italy

Email: tresoldi.moreno@hsr.it

Villa Paolo

Department of Emergency, ASST FBF - Sacco, Ospedale L.Sacco, Milan, Italy

Email: villa.paolo@hsacco.it

Greco Stefano

Departement of Anesthesia and Intensive Care, ASST Valle Olona, Ospedale Busto Arsitio, Busto Arsitio, Italy

Email: sgreco@aobusto.it

Bonfanti Paolo

Infectious diseases, Fondazione IRCCS San Gerardo dei Tintori, Monza, Italy

Email: [paolo.bonfanti@unimib.it](mailto:paolo.bonfanti@unimib.it)

Spitoni Maria Grazia

ASST Bergamo ovest, Ospedale di Treviglio e Caravaggio, Treviglio, Italy

Email: mariagrazia.spitoni@libero.it

Vesconi Sergio

Department of Anesthesia and Intensive Care, ASST GOM Niguarda Ca’ Granda, Milan Italy

Email: [sergiovesconi@tin.it](mailto:sergiovesconi@tin.it)

Caironi Pietro

Department of Anesthesia and Intensive Care, AOU S. Luigi Gonzaga, Università degli Studi di Torino, Orbassano, Italy

Email: [pietro.caironi@unito.it](mailto:pietro.caironi@unito.it)

Fumagalli Roberto

School of Medicine and Surgery, University of Milano-Bicocca, Monza, Italy

Department of Anesthesia and Intensive Care, ASST GOM Niguarda Ca’ Granda, Milan Italy

Email: [roberto.fumagalli@unimib.it](mailto:roberto.fumagalli@unimib.it)

**Methods**

*Study design*

This is a prospective multicenter before-and-after cohort study conducted as part of a quality improvement program (QIP) of Lombardy Region (Northern Italy), including 12 Emergency Departments (EDs) and 39 Medical Wards (MWs) of 12 University and Non-Academic Hospitals, under the coordination of ASST GOM Niguarda Ca’ Granda (Milan, Italy). We enclose below data on ED and MW with number of beds for each Hospital enrolled in the study:

| Hospital | ED  Level | ED  Admission /Year 2010 | ED Admission /  Year 2022 | Hospital  Beds | MW beds enrolled |
| --- | --- | --- | --- | --- | --- |
| Niguarda | II | 87013 | 90647 | 1167 | 229 |
| Legnano | II | 58311 | 67979 | 550 | 91 |
| Busto | I | 50242 | 49783 | 661 | 123 |
| Bergamo | II | 80429 | 86383 | 1024 | 104 |
| Sacco | I | 51700 | 37688 | 507 | 58 |
| Varese | II | 68000 | 67472 | 600 | 119 |
| San Raffaele | II | 63000 | 69012 | 1373 | 53 |
| Policlinico | II | 52000 | 86885 | 910 | 30 |
| Treviglio | I | 52270 | 44530 | 407 | 100 |
| Desio | I | 64344 | 59702 | 120 | 54 |
| Pavia | II | 65000 | 86237 | 1024 | 111 |
| Lecco | I | 60583 | 61665 | 1000 | 88 |

The quality improvement program included the implementation of an educational intervention for medical staff and nurses, as well as an organizational intervention to point out the resources available in each hospital for the management of septic patients, and to further face the barriers to the implementation. Patients with a clinical diagnosis of severe sepsis or septic shock identified during the study periods in the EDs or MWs were included. We enrolled patients with diagnosis of sepsis or septic shock, according to the definitions of the 2001 International Sepsis Definitions Conference (Sepsis-2) [1], out of ICU from May to November 2011 (before-QIP cohort) and from August 2012 to June 2013 (after-QIP cohort). The Third International Consensus Definition for Sepsis and Septic Shock (Sepsis-3) [2] was not employed as they were released after the QIP was concluded. A post-hoc adjudication approach was not deemed to be accurate. After the implementation period (from August 2012 to June 2013), the same data were recorded in a new cohort of patients with severe sepsis or septic shock.

*Description of the Quality Improvement Program (QIP): educational and re-organizational intervention.*

The quality improvement program (QIP) started in Lombardy Region in 2011, and has been active since then. The QIP described below aimed to improve the quality of sepsis care, to implement QI teams in the participating hospital and to evaluate outcome by applying a series of educational and organizational interventions [3]. Both educational and re-organizational intervention were directed to EDs/MWs medical staff and nurses with the aim to increase sepsis awareness and to increase the standard of care in sepsis and septic shock. A local multidisciplinary QI team was involved in the educational and reorganizational interventions. The educational intervention consisted in both an educational course for both physicians and nurses of EDs and MWs of the participating centers. Five hundred medical doctors (78% of the staff) and 1159 nurses (80% of the staff) were respectively, trained by means of 49 and 50 courses.

QIP was based on a “waterfall model” of training: the coordinating center was responsible for the education of the local “QI trainers”. Local QI trainers were identified in each center and were involved in the educational process of their own hospital staff of ED and MWs. Pocket cards and posters summarizing information on sepsis diagnosis and treatment were available in each hospital (Suppl. 1). Further details on the program are described in the online data supplement (Suppl. 2).

The organizational intervention was designed to facilitate the implementation of evidence-based guidelines by the local QI team and health professionals. An organizational logistic check list (OCL) was provided to each participating center as a guide to identify the resources /tools available locally for the management of septic patients. Each participating center was asked to face independently the barriers to implementation detected during the study period. Regular meetings were organized to provide the involved investigators feedback information and keep them up to date as follows:

- 2012/09: Meeting to present the first report on the single center and overall activity

- 2013/01: e-mailing a specific report to each center

- 2013/02: Multicenter Meeting to present new 2012 SSC Guidelines and state of the study

- 2013/04: 2013 ISICEM Congress: e-poster presenting the Human and Organizational resource available in each hospital before QIP;

- 2013/05: 2013 SMART Congress, Milan: presentation of preliminary results;

- 2013/05: e-mailing a confidential report to Regione Lombardia

- 2013/09: 2013 World Sepsis Day, Milan: presentation of preliminary results;

- 2014/01: Multicenter Meeting to present preliminary data;

- 2016/09: Multicenter Meeting to present conclusive data.

*Study of intervention*

During the study period, demographic data type of participating units (ED or MW), timing and date of suspicion of sepsis, sepsis severity, infection site, as well as compliance data related to the application of S6 bundles (including timing/ mode/ reason - in case of not application) and to other clinical interventions were recorded for the two cohorts of patients: the before-QIP cohort (enrolled from May to November 2011) and the after-QIP cohort (enrolled from August 2012 to June 2013).

Data were recorded on a preformed clinical checklist (Supplemental Figure 2) by the medical and nursing staff of each participating center. Data on SOFA score and comorbidities were retrieved retrospectively. Data on hospital mortality rate were obtained from administrative registries of each participating centers. Data recorded in the check-lists were periodically entered into electronic case report form (CRF) at the Coordinating Center. Data on the logistical-organizational resources of each participating center were registered at the beginning and at the end of the study.

Collected data underwent a double quality control. The first one, was performed by three independent investigators at the Promoting Center of Niguarda Hospital. A second quality control of collected data was performed by two independent Statisticians.

*Definitions*

According to the 2001 International Sepsis Definitions Conference [1] and 2008 SSC [5], we defined as “Severe Sepsis”, the presence of a suspected infection and at least one sepsis-related organ dysfunction, as defined above, and as “Septic Shock”, the presence of Severe Sepsis associated with the persistence of hypotension requiring vasopressor administration despite adequate fluid resuscitation or serum lactate level ≥ 4 mmol/L.

According to the 2008 edition of the Surviving Sepsis Campaign guidelines - which were followed during the study course - we considered an adequate fluid resuscitation the administration of 20 ml/kg of crystalloid fluids in case of septic patient with hypotension (systolic blood pressure < 90 mmHg) or Lactate level ≥ 4 mmol/L; the administration of 500 ml of crystalloid fluid in case of septic patient with other organ dysfunctions other than hypotension and/or Lactate level ≥ 4 mmol/L [5] We allowed a 20% reduction in fluid resuscitation for septic patients suffering cardiomyopathy or renal failure at the hospital admission. When not reported on patient check-list, we assumed 60 kg and 70 kg of body weight for female and male patients respectively.

According to 2008 SSC guidelines, at least two blood cultures were obtained before antibiotics with at least one drawn percutaneously and one drawn through each vascular access device, unless the de- vice was recently (< 48 hrs) inserted. Volume of blood drawn with the culture tube should be more than 10 mL. Intravenous antibiotic therapy should be started as early as possible and within the first hour of recognition of septic shock or severe sepsis.

**Supplemental Figure 1.** Organizational logistic checklist.

Hospital _________________________ Ward _____________________

ORGANISATIONAL & LOGISTIC CHECK-LIST (OLC)

1) In your hospital, do you have a *Diagnostic and therapeutic protocol for septic patient management*?

YES ⁫ NO ⁫

If yes, a) Has an educational sharing program been implemented? YES ⁫ NO ⁫

b) How could the protocol be reviewed?

- paper format YES ⁫ NO ⁫ ⁫

- online format ⁫ YES ⁫ NO ⁫

2) In your hospital, is an *Early Warning Score or similar* being used for an early diagnosis and initial management of sepsis?

YES ⁫ NO ⁫

If yes, In which wards? ED MW

What kind of score? …………………………….

Is the score repeatedly evaluated at set scheduled times? YES ⁫ NO ⁫

Is the nurse staff allowed to carry out some interventions? YES ⁫ NO ⁫

3) In your hospital, do you have a *Sepsis team or a Rapid response team*?

YES ⁫ NO ⁫

4) In your hospital, is the *Micro-Lab* able to process blood cultures 24/7?

YES ⁫ NO

a) Is a blood colture execution protocol set up? YES ⁫ NO

b) Is your Micro-Lab able to give a preliminary response within 48 hours? YES ⁫ NO ⁫

5) In your hospital is the *biochemistry-lab*  able to process urgent exams 24/7?

YES ⁫ NO

6) In your hospital, is it possible to have an urgent *Serum Lactate* measurement 24/7 in all wards (through a point of care and/or biochemistry-lab)?

⁫ YES ⁫ NO

a) Time to result in ED < 60 minutes ⁫ YES ⁫ NO

b) Time to results in MW < 60 minutes YES ⁫ NO

7) In your hospital, is it possible to insert a *Central Venous Catheter* 24/7?

YES ⁫ NO

8) In your hospital, do you have any *Hospital Antibiotic therapy guidelines*?

YES ⁫ NO

If yes, a) Is an educational sharing program set up? YES ⁫ NO ⁫

b) How could the protocol be reviewed?

- paper format YES ⁫ NO ⁫ ⁫

- online format ⁫ YES ⁫ NO ⁫

9) Is *surgical team* ready available for professional advice or for infectious source eradication?

YES ⁫ NO

10) Is an *Infectious disease team (IDT)* ready and available for professional advice?

YES ⁫ NO

If yes, is the IDT routinely involved in any case of sepsis or septic shock? YES NO

11) *Infective source eradication*

a) Is an operating room available 24/7? YES NO

b) Does an infective source eradication protocol exist? YES NO

c) Is an intervention radiology lab available 24/7? YES NO

d) Is a meningitides infection protocol set up? YES NO

e) Is a CR-BSI management protocol set up? YES NO

12) Is an *Imaging reporting service* available 24/7?

YES ⁫ NO

**Supplemental Figure 2.** Clinical checklist.


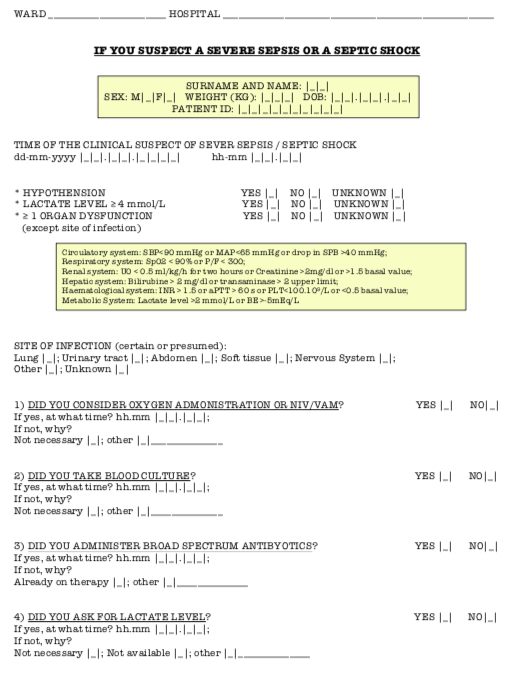


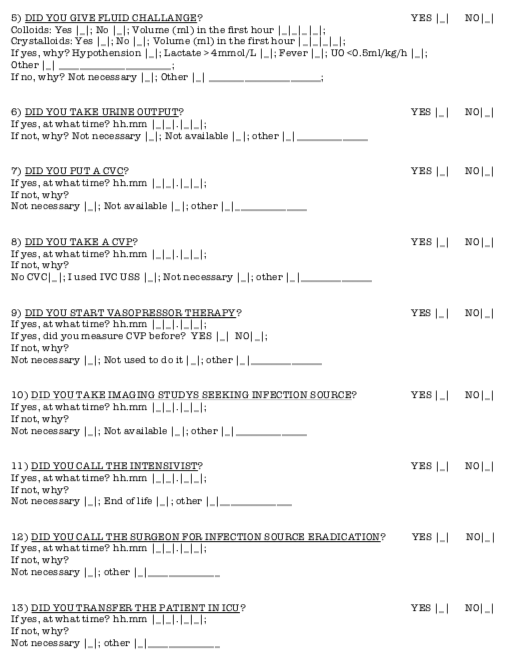


**Supplemental Figure 3.** Flow of the before and after quality improvement program implementation. Not classified patients were the ones with missing data that did not allow a proper definition of severe sepsis and septic shock. However, not classified were included as part of the intention to treat analysis.


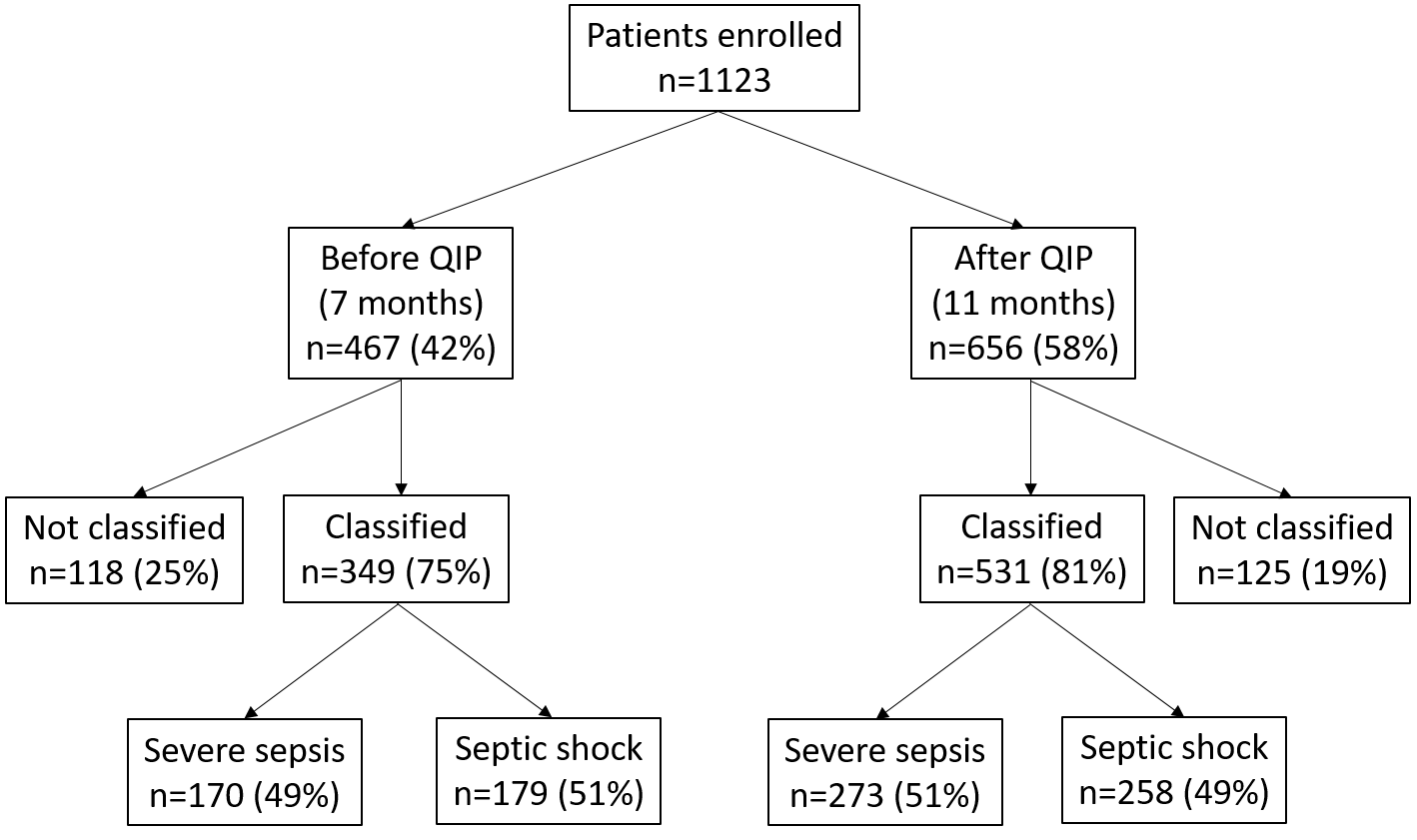


**Supplemental Table 1.** Analysis of adherence to the sepsis’ six procedures applied within the first 3 hours between the two study groups (after *versus* before QIP). **p* < 0.05.

| ***Procedures applied within 3hr*** | **Before QIP** | **After QIP** | **P value** |
| --- | --- | --- | --- |
| Evaluation of need  for O_2_ /NIMV/MV – no. (%), n=861 | 369/393 (93.9%) | 430/468 (91.9%) | 0.315 |
| *time elapsed after enrollment – min, n=799* | 0 [0-5] | 0 [0-10] | 0.007* |
| Blood culture – no. (%), n=1017 | 298/405 (73.6%) | 470/612 (76.8%) | 0.274 |
| *time elapsed after enrollment – min, n=768* | 10 [0-40] | 10 [0-40] | 0.592 |
| Antibiotic therapy – no. (%), n=986 | 298/392 (76.0%) | 478/594 (80.5%) | 0.112 |
| *time elapsed after enrollment – min, n=776* | 45 [15-98.8] | 45 [10-90] | 0.230 |
| Lactate measurement – no. (%), n=1054 | 248/433 (57.3%) | 466/621 (75.0%) | <0.001* |
| *time elapsed after enrollment – min, n=714* | 0 [0-17.8] | 0 [0-19.2] | 0.936 |
| Fluid administration – no. (%), n=1047 | 194/436 (44.5%) | 296/611 (48.4%) | 0.230 |
| Urinary output measurement – no. (%), n=984 | 276/387 (71.3%) | 443/597 (74.2%) | 0.356 |
| *time elapsed after enrollment – min, n=719* | 6.5 [0-30] | 5 [0-30] | 0.286 |
|  |  |  |  |
| No. of procedures adequately applied  – no. (%), n=644 |  |  |  |
| 0 procedure | 1/267 (0.3%) | 0/377 (0.0%) | 0.013* |
| 1 procedures | 4/267 (1.4%) | 4/377 (1.1%) |  |
| 2 procedures | 15/267 (5.6%) | 9/377 (2.3%) |  |
| 3 procedures | 31/267 (11.6%) | 23/377 (6.1%) |  |
| 4 procedures | 42/267 (15.7%) | 61/377 (16.1%) |  |
| 5 procedures | 75/267 (28.1%) | 99/377 (26.2%) |  |
| All procedures (all 6) – no. (%) | 99/267 (37.1%) | 181/377 (48.0%) |  |

**Supplemental Table 2. Post-hoc analysis – heterogeneity according to the presence of shock, the lactate level, the presence of hypotension, the type of admission, the type of discharge and the tertiles of SOFA.**

Analysis of the possible interaction between the presence of shock, the lactate level, the presence of hypotension, the type of admission, the type of discharge, the tertiles of SOFA and six-bundle adherence within 1^st^ hour. **p* < 0.05.

|  | **Adherence to all six procedures within 1^st^hr** | | | | |
| --- | --- | --- | --- | --- | --- |
| **Shock yes/no** | **All** | **Before QIP** | **After QIP** | **Relative**  **Risk**  **(95% CI)** | **P value**  *(control vs. interv.)* |
| *Severe sepsis without shock* | 67/396 (16.9%) | 15/157 (9.6%) | 52/239 (21.8%) | 2.28 (1.33;3.90) | 0.002* |
| *Septic shock* | 86/384 (22.4%) | 34/157 (21.7%) | 52/227 (22.9%) | 1.06 (0.72;1.55) | 0.772 |
| *Interaction* | — | — | — | — | 0.025* |
| **Lactate** | All | Before QIP | After QIP | Relative  Risk  (95% CI) | P value  (control vs. interv.) |
| *Lactate Yes* | 74/320 (23.1%) | 30/129 (23.3%) | 44/191 (23.0%) | 0.99 (0.66;1.49) | 0.964 |
| *Lactate No* | 80/479 (16.7%) | 19/192 (9.9%) | 61/287 (21.3%) | 2.15 (1.33;3.48) | 0.001* |
| *Interaction* |  |  |  |  | 0.020* |
| **Hypotension** | All | Before QIP | After QIP | Relative  Risk  (95% CI) | P value  (control vs. interv.) |
| *Hypotension No* | 58/421 (13.8%) | 18/169 (10.7%) | 40/252 (15.9%) | 1.49 (0.89;2.51) | 0.128 |
| *Hypotension Yes* | 104/586 (17.7%) | 33/256 (12.9%) | 71/330 (21.5%) | 1.67 (1.14;2.44) | 0.007* |
| *Interaction* |  |  |  |  | 0.682 |
| **Admission from** | All | Before QIP | After QIP | Relative  Risk  (95% CI) | P value  (control vs. interv.) |
| *Medical Ward* | 31/402 (7.7%) | 8/174 (4.6%) | 23/228 (10.1%) | 2.19 (1.01;4.79) | 0.041* |
| *ED* | 133/612 (21.7%) | 44/255 (17.3%) | 89/357 (24.9%) | 1.44 (1.05;2.00) | 0.023* |
| *Interaction* |  |  |  |  | 0.426 |
| **SOFA tertiles** | All | Before QIP | After QIP | Relative  Risk  (95% CI) | P value  (control vs. interv.) |
| *Tertile 1* | 63/314 (20.1) | 20/126 (15.9%) | 43/188 (22.9%) | 1.44 (0.89;2.33) | 0.129 |
| *Tertile 2* | 26/180 (14.4) | 7/76 (9.2%) | 19/104 (18.3%) | 1.98 (0.88;4.48) | 0.088 |
| *Tertile 3* | 31/204 (15.2) | 12/94 (12.8%) | 19/110 (17.3%) | 1.35 (0.69;2.64) | 0.371 |
| *Interaction* |  |  |  |  | 0.772 |

**Supplemental Table 3. Post-hoc analysis – heterogeneity according to the presence of shock, the lactate level, the presence of hypotension, the type of admission, the type of discharge and the tertiles of SOFA.**

Analysis of the possible interaction between the presence of shock, the lactate level, the presence of hypotension, the type of admission, the type of discharge, the tertiles of SOFA and six-bundle adherence within the entire study period. **p* < 0.05.

|  | **Adherence to all six procedures within the study period** | | | | |
| --- | --- | --- | --- | --- | --- |
| **Shock yes/no** | **All** | **Before QIP** | **After QIP** | **Relative**  **Risk**  **(95% CI)** | **P value**  *(control vs. interv.)* |
| *Severe sepsis without shock* | 220/433 (50.8%) | 61/165 (37%) | 159/268 (59.3%) | 1.60 (1.28;2.00) | <0.001* |
| *Septic shock* | 296/430 (68.8%) | 121/176 (68.8%) | 175/254 (68.9%) | 1.00 (0.88;1.14) | 0.974 |
| *Interaction* | — | — | — | — | 0.002* |
| **Lactate** | All | Before QIP | After QIP | Relative  Risk  (95% CI) | P value  (control vs. interv.) |
| *Lactate Yes* | 244/353 (69.1%) | 99/141 (70.2%) | 145/212 (68.4%) | 0.97 (0.85;1.12) | 0.717 |
| *Lactate No* | 273/527 (51.8%) | 81/207 (39.1%) | 192/320 (60.0%) | 1.53 (1.27;1.86) | <0.001* |
| *Interaction* |  |  |  |  | 0.002* |
| **Hypotension** | All | Before QIP | After QIP | Relative  Risk  (95% CI) | P value  (control vs. interv.) |
| *Hypotension No* | 200/451 (44.3%) | 67/181 (37.0%) | 133/270 (49.3%) | 1.33 (1.06;1.67) | 0.010* |
| *Hypotension Yes* | 364/644 (56.5%) | 130/273 (47.6%) | 234/371 (63.1%) | 1.32 (1.14;1.53) | <0.001* |
| *Interaction* |  |  |  |  | 0.614 |
| **Admission from** | All | Before QIP | After QIP | Relative  Risk  (95% CI) | P value  (control vs. interv.) |
| *Medical Ward* | 171/437 (39.1%) | 49/184 (26.6%) | 122/253 (48.2%) | 1.81 (1.38;2.38) | <0.001* |
| *ED* | 396/666 (59.5%) | 149/274 (54.4%) | 247/392 (63%) | 1.16 (1.02;1.32) | 0.026* |
| *Interaction* |  |  |  |  | 0.026* |
| **SOFA tertiles** | All | Before QIP | After QIP | Relative  Risk  (95% CI) | P value  (control vs. interv.) |
| *Tertile 1* | 189/344 (54.9) | 62/134 (46.3%) | 127/210 (60.5%) | 1.31 (1.06;1.62) | 0.010* |
| *Tertile 2* | 98/194 (50.5) | 32/82 (39%) | 66/112 (58.9%) | 1.51 (1.11;2.06) | 0.006* |
| *Tertile 3* | 124/218 (56.9) | 49/99 (49.5%) | 75/119 (63%) | 1.27 (1.00;1.62) | 0.045* |
| *Interaction* |  |  |  |  | 0.783 |

**Supplemental Table 4.** Analysis of hospital mortality as comparison of *intervention period vs. control period by RR (95%CI).*

| ***Variables*** | **Before QIP** | **After QIP** | **Relative Risk (95% CI)** | **P value** |
| --- | --- | --- | --- | --- |
| Hospital mortality – no. (%), n=1089 | 170/448 (37.9%) | 208/641 (32.4%) | 0.92 (0.84-1.00) | 0.062 |

**Supplemental Table 5. Post-hoc analysis – heterogeneity according to admission characteristics.** Analysis of the possible interaction between the presence of shock, levels of lactates, hypotension, type of admission, type of discharge, SOFA tertiles and hospital mortality.

**p* < 0.05.

|  | **Hospital mortality** | | | | |
| --- | --- | --- | --- | --- | --- |
| **Shock** | **All** | **Before QIP** | **After QIP** | **Relative Risk**  **(95% CI)** | **P value**  *(control vs. interv.)* |
| Severe sepsis without shock | 132/437 (30.2%) | 63/165 (38.2%) | 69/272 (25.4%) | 0.66 (0.50;0.88) | 0.005* |
| Septic shock | 179/422 (42.4%) | 73/172 (42.4%) | 106/250 (42.4%) | 1.00 (0.80;1.25) | 0.993 |
| *Interaction* | — | — | — | — | 0.042* |
| **Lactate** |  |  |  |  |  |
| Lactate Yes | 153/346 (44.2%) | 59/138 (42.8%) | 94/208 (45.2%) | 1.06 (0.83;1.35) | 0.655 |
| Lactate No | 154/529 (29.1%) | 75/206 (36.4%) | 79/323 (24.5%) | 0.67 (0.52;0.87) | 0.003* |
| *Interaction* |  |  |  |  | 0.023* |
| **Hypotension** |  |  |  |  |  |
| Hypotension No | 115 (25.8%) | 43/179 (24%) | 72/266 (27.1%) | 1.13 (0.81;1.56) | 0.472 |
| Hypotension Yes | 260 (40.9%) | 126/265 (47.5%) | 134/371 (36.1%) | 0.76 (0.63;0.91) | 0.004* |
| *Interaction* |  |  |  |  | 0.022* |
| **Admission from** |  |  |  |  |  |
| Medical Ward | 153/428 (35.7%) | 72/183 (39.3%) | 81/245 (33.1%) | 0.84 (0.65;1.08) | 0.180 |
| ED | 225/661 (34%) | 98/265 (37%) | 127/396 (32.1%) | 0.87 (0.70;1.07) | 0.192 |
| *Interaction* |  |  |  |  | 0.835 |
| **SOFA tertiles** |  |  |  |  |  |
| Tertile 1 | 79/350 (22.6) | 37/134 (27.6) | 42/216 (19.4) | 0.70 (0.48-1.04) | 0.076 |
| Tertile 2 | 68/200 (34.0) | 29/85 (34.1) | 39/115 (33.9) | 0.99 (0.67-1.47) | 0.976 |
| Tertile 3 | 101/220 (45.9) | 55/100 (55.0) | 46/120 (38.3) | 0.70 (0.52-0.93) | 0.014* |
| *Interaction* |  |  |  |  | 0.258 |

**Supplemental Table 6. Analysis of association between interventions and risk of death.**

Analysis of the association between the adherence to each single interventions (applied either within the first hour **(A)** or within the study period **(B)**) and the reduction of the risk of death (hospital mortality) at univariate analysis. **p* < 0.05.

| **A - Procedures applied**  **within 1^st^hr** | **Category** | **Death**  **(no – %)** | **OR**  **95% CI** | **p-value** |
| --- | --- | --- | --- | --- |
|  |  |  |  |  |
| Mechanical ventilation | Compliant | 268/732 (36.6%) | 1.24 (0.80;1.95) | 0.332 |
|  | Non-compliant | 33/104 (31.7%) | — | — |
| Blood culture | Compliant | 190/645 (29.5%) | 0.50 (0.38;0.66) | <0.001* |
|  | Non-compliant | 154/340 (45.3%) | — | — |
| Antibiotic therapy | Compliant | 160/504 (31.7%) | 0.76 (0.58;1.00) | 0.046* |
|  | Non-compliant | 172/454 (37.9%) | — | — |
| Lactate measurement | Compliant | 220/639 (34.4%) | 0.98 (0.75;1.28) | 0.879 |
|  | Non-compliant | 134/384 (34.9%) | — | — |
| Adequate fluid therapy | Compliant | 161/478 (33.7%) | 0.90 (0.69;1.16) | 0.407 |
|  | Non-compliant | 196/542 (36.2%) | — | — |
| Urinary output measurement | Compliant | 228/622 (36.7%) | 1.36 (1.02;1.81) | 0.034* |
|  | Non-compliant | 100/335 (29.9%) | — | — |
|  |  |  |  |  |
| No. of procedures adequately applied – no. (%) |  |  |  |  |
| 0-2 procedures |  | 37/97 (38.1%) | — | 0.003* |
| 3 procedures (Ref. 0-2 procedures) |  | 51/99 (51.5%) | 1.72 (0.97;3.05) |  |
| 4 procedures (Ref. 0-2 procedures) |  | 54/158 (34.2%) | 0.84 (0.5;1.43) |  |
| 5 procedures (Ref. 0-2 procedures) |  | 40/149 (26.8%) | 0.60 (0.34;1.03) |  |
| 6 procedures (Ref. 0-2 procedures) |  | 36/100 (36%) | 0.91 (0.51;1.63) |  |
| *P for trend* |  |  | 0.62 (0.15;2.24) | 0.031* |

| **B - Procedures applied**  **within entire study period** | **Category** | **Death**  **(no – %)** | **OR**  **95% CI** | **p-value** |
| --- | --- | --- | --- | --- |
|  |  |  |  |  |
| Mechanical ventilation | Compliant | 364/1044 (34.9%) | 0.90 (0.45;1.87) | 0.781 |
|  | Non-compliant | 13/35 (37.1%) | — | — |
| Blood culture | Compliant | 293/914 (32.1%) | 0.49 (0.35;0.68) | <0.001* |
|  | Non-compliant | 83/169 (49.1%) | — | — |
| Antibiotic therapy | Compliant | 341/994 (34.3%) | 0.82 (0.52;1.31) | 0.401 |
|  | Non-compliant | 33/85 (38.8%) | — | — |
| Lactate measurement | Compliant | 290/844 (34.4%) | 0.91 (0.68;1.24) | 0.559 |
|  | Non-compliant | 87/239 (36.4%) | — | — |
| Adequate fluid therapy | Compliant | 340/978 (34.8%) | 1.05 (0.68;1.64) | 0.829 |
|  | Non-compliant | 33/98 (33.7%) | — | — |
| Urinary output measurement | Compliant | 339/897 (37.8%) | 2.25 (1.55;3.34) | <0.001* |
|  | Non-compliant | 38/179 (21.2%) | — | — |
|  |  |  |  |  |
| No. of procedures adequately applied – no. (%) |  |  |  |  |
| 0-3 procedures |  | 24/70 (34.3%) | — | 0.854 |
| 4 procedures (Ref. 0-3 procedures) |  | 37/111 (33.3%) | 0.96  (0.51;1.82) |  |
| 5 procedures (Ref. 0-3 procedures) |  | 114/307 (37.1%) | 1.13 (0.66;1.98) |  |
| 6 procedure (Ref. 0-3 procedures) |  | 189/546 (34.6%) | 1.01 (0.60;1.74) |  |
| *P for trend* |  |  | 0.00 (0.00;0.00) | 0.991 |

**Supplemental Table 7.** Differences between groups of patients managed after *versus* before QIP on hemodymanic lines and pressors management, use of diagnostic imaging, specialist calls, and transfer to ICU. **p* < 0.05.

|  | **Before QIP (%)** | **After QIP (%)** | p |
| --- | --- | --- | --- |
| CVC placement, n=1111 | 105/460 (22.8) | 110/651 (16.9) | 0.017* |
| CVP measurement, n=1107 | 97/459 (21.1) | 156/648 (24.1) | 0.282 |
| Vasopressor use, n=1108 | 138/458 (30.1) | 169/650 (26.0) | 0.149 |
| Diagnostic imaging use, n=1110 | 409/461 (88.7) | 563/649 (86.7) | 0.374 |
| ICU consultant call, n=1118 | 134/465 (28.8) | 189/653 (28.9) | 1.000 |
| Surgeon call, n=1105 | 52/454 (11.5) | 78/651 (12.0) | 0.863 |
| Transfer to the ICU, n=1107 | 47/459 (10.2) | 54/648 (8.3) | 0.327 |

**Supplemental Table 8.** Differences in organizational resources available for septic patient management between after *versus* before QIP (N=12 corresponding to the number of enrolled centers). **p* < 0.05.

| **Resource** | **Availability** | | **McNemar**  **p-value** |
| --- | --- | --- | --- |
|  | **Before QIP (%)** | **After QIP (%)** |  |
| Diagnostic and Therapeutic protocol for septic patient management | 8.3 | 50 | 0.073 |
| Early warning score or similar for diagnosis and management | 50 | 80 | / |
| Data available, n | 2 | 5 |  |
| Sepsis team or rapid response system | 25 | 91.7 | 0.013 |
| Microbiology laboratory 7 days a week able to process blood cultures | 54.5 | 83.3 | 0.248 |
| Not available, n | 1 |  |  |
| - Hospital blood cultures execution protocol | 91.7 | 100 | 1 |
| - MicroLab preliminary response in ≤48 h | 100 | 100 | 1 |
| Biochemistry laboratory 7 days a week open | 100 | 100 | 1 |
| Lactate dosage 24 h a day | 90.9 | 100 | 1 |
| Not available, n | 1 |  |  |
| - Time for result in Emergency Department: ≤ 60’ | 83.3 | 100 | 0.48 |
| Time for result in Medical Wards: ≤ 60’ | 58.3 | 72.7 | 1 |
| Not available, n |  | 1 |  |
| Central venous catheter insertion 24 h a day | 91.7 | 83.3 | 1 |
| Hospital antibiotic therapy guide lines | 9 | 41.7 | 0.074 |
| Surgical team professional advice | 100 | 100 | 1 |
| Infection diseases team (IDT) professional advice | 100 | 100 | 1 |
| Not available, n | 3 | 2 |  |
| - ID involvement in any case of severe sepsis or septic shock | 25 | 25 | 1 |
| Infective source eradication: |  |  |  |
| - Operating room 24h a day available | 100 | 100 | 1 |
| - Infection source eradication protocols | 8.3 | 8.3 | 1 |
| - Intervention radiology 24h a day available | 50 | 66.7 | 0.48 |
| - Meningitidis infection protocol | 41.7 | 54.5 | 1 |
| Not available, n |  | 1 |  |
| - CVC infection protocol | 58.3 | 81.8 | 0.248 |
| Not available, n |  | 1 |  |
| Imaging reporting service 7 days a week open | 41.7 | 45.5 | 1 |
| Not available, n | 1 |  |  |

**REFERENCES**

1. Levy MM, Fink MP, Marshall JC, et al. 2001 SCCM/ESICM/ACCP/ATS/SIS International Sepsis Definitions Conference. *Crit Care Med*. 2003;31(4):1250-1256. doi:10.1097/01.CCM.0000050454.01978.3B
2. Singer M, Deutschman CS, Seymour CW, et al. The Third International Consensus Definitions for Sepsis and Septic Shock (Sepsis-3). *JAMA*. 2016;315(8):801-810. doi:10.1001/jama.2016.0287
3. Schouten LM, Hulscher ME, van Everdingen JJ, Huijsman R, Grol RP. Evidence for the impact of quality improvement collaboratives: systematic review. *BMJ*. 2008;336(7659):1491-1494. doi:10.1136/bmj.39570.749884.BE
4. Robson WP, Daniel R. The Sepsis Six: helping patients to survive sepsis. *Br J Nurs*. 2008;17(1):16-21. doi:10.12968/bjon.2008.17.Sup1.28145
5. Dellinger RP, Levy MM, Carlet JM, et al. Surviving Sepsis Campaign: international guidelines for management of severe sepsis and septic shock: 2008 [published correction appears in Crit Care Med. 2008 Apr;36(4):1394-6]. *Crit Care Med*. 2008;36(1):296-327. doi:10.1097/01.CCM.0000298158.12101.41
6. Agresti A, Min Y. Unconditional small-sample confidence intervals for the odds ratio. *Biostatistics*. 2002;3(3):379-386. doi:10.1093/biostatistics/3.3.379
7. Breslow NE, Day NE. Statistical methods in cancer research. Volume I - The analysis of case-control studies. *IARC Sci Publ*. 1980;(32):5-338.
8. Fitzmaurice GM, Garrett M, Nan M. Laird, and James H. Ware. *Applied longitudinal analysis*. Vol. 998. John Wiley & Sons, 2012.
9. R Core Team (2018). R: A language and environment for statistical computing. R Foundation for Statistical Computing, Vienna, Austria. URL https://www.R-project.org.
